# EV-101: A Phase I Study of Single-Agent Enfortumab Vedotin in Patients With Nectin-4–Positive Solid Tumors, Including Metastatic Urothelial Carcinoma

**DOI:** 10.1200/JCO.19.02044

**Published:** 2020-02-07

**Authors:** Jonathan Rosenberg, Srikala S. Sridhar, Jingsong Zhang, David Smith, Dean Ruether, Thomas W. Flaig, Joaquina Baranda, Joshua Lang, Elizabeth R. Plimack, Randeep Sangha, Elisabeth I. Heath, Jamie Merchan, David I. Quinn, Sandy Srinivas, Matthew Milowsky, Chunzhang Wu, Elaina M. Gartner, Peiying Zuo, Amal Melhem-Bertrandt, Daniel P. Petrylak

**Affiliations:** ^1^ Memorial Sloan Kettering Cancer Center, New York, NY; ^2^ Princess Margaret Cancer Centre, University Health Network, Toronto, Ontario, Canada; ^3^ H. Lee Moffitt Cancer Center and Research Institute, Tampa, FL; ^4^ University of Michigan, Ann Arbor, MI; ^5^ Tom Baker Cancer Centre, Calgary, Alberta, Canada; ^6^ University of Colorado Comprehensive Cancer Center, Aurora, CO; ^7^ University of Kansas Cancer Center, Fairway, KS; ^8^ University of Wisconsin Carbone Cancer Center, Madison, WI; ^9^ Fox Chase Cancer Center, Philadelphia, PA; ^10^ Cross Cancer Institute, Edmonton, Alberta, Canada; ^11^ Barbara Ann Karmanos Cancer Institute, Wayne State University, Detroit, MI; ^12^ University of Miami, Miami, FL; ^13^ University of Southern California Norris Comprehensive Cancer Center, Los Angeles, CA; ^14^ Stanford University, Stanford, CA; ^15^ UNC Lineberger Comprehensive Cancer Center, Chapel Hill, NC; ^16^ Astellas Pharma, Northbrook, IL; ^17^ Seattle Genetics, Bothell, WA; ^18^ Yale School of Medicine, New Haven, CT

## Abstract

**PURPOSE:**

To assess the safety/tolerability and antitumor activity of enfortumab vedotin (EV), a novel investigational antibody-drug conjugate that delivers the microtubule-disrupting agent, monomethyl auristatin E, to cells that express Nectin-4.

**METHODS:**

EV-101 is a phase I dose escalation/expansion study that enrolled patients with Nectin-4–expressing solid tumors (eg, metastatic urothelial carcinoma [mUC]) who progressed on ≥ 1 prior chemotherapy regimen and/or programmed death-1 receptor/programmed death ligand-1 [PD-(L)1] inhibitor, including a cohort of patients with mUC who received prior anti–PD-(L)1 therapy. Patients received escalating doses of EV up to 1.25 mg/kg on days 1, 8, and 15 of every 28-day cycle. Primary objectives were evaluation of safety/tolerability and pharmacokinetics; antitumor activity was a secondary objective.

**RESULTS:**

Enrolled patients with mUC (n = 155) were heavily pretreated, with 96% having prior platinum-based chemotherapy and 29% receiving ≥ 3 lines of prior treatment. Maximum tolerated dose of EV was not established; however, the recommended phase II dose was identified as 1.25 mg/kg. Rash, peripheral neuropathy, fatigue, alopecia, and nausea were the most common treatment-related adverse events (TRAEs); the most common TRAEs were grade 1-2 in severity. Among the 112 patients with mUC treated with single-agent EV 1.25 mg/kg, the investigator-assessed confirmed objective response rate (ORR) was 43%, and duration of response was 7.4 months. Median overall survival (OS) was 12.3 months, and the OS rate at 1 year was 51.8%. Similar ORR and estimated median OS were observed in patients ≥ 75 years of age with and without prior anti–PD-(L)1 treatment, liver metastases, or upper-tract disease.

**CONCLUSION:**

Single-agent EV was generally well tolerated and provided clinically meaningful and durable responses in patients with mUC; survival data are encouraging. A pivotal phase II and a confirmatory phase III study are ongoing.

## INTRODUCTION

Nectin-4 is a type 1 transmembrane protein and member of a family of related immunoglobulin-like adhesion molecules implicated in cell-cell adhesion.^1^ Nectin-facilitated adhesion supports several biologic processes, such as immune modulation, host-pathogen interaction, and immune evasion.^1^ Nectin-4 is highly expressed in cancer cells, particularly in urothelial carcinomas (UCs), with moderate expression observed in normal human skin.^2-5^ Enfortumab vedotin (EV; previously known as ASG-22CE) is a novel, fully humanized, monoclonal antibody-drug conjugate (ADC) that delivers a microtubule-disrupting agent, monomethyl auristatin E (MMAE), to cells that express Nectin-4. EV selectively binds to Nectin-4–expressing cells, initiating internalization of the ADC-Nectin-4 complex and proteolytic cleavage of the conjugated MMAE, disrupting microtubule networks, and resulting in apoptotic death.^2^

Currently, a high unmet medical need exists for effective and tolerable treatments in patients with metastatic UC (mUC). Standard first-line therapy consists of cisplatin-based combination chemotherapy with a 5-year survival rate of < 5%.^6-8^ Moreover, up to 50% of patients with UC are not eligible to receive cisplatin-based chemotherapy because of comorbidities such as renal dysfunction, heart failure, or low Eastern Cooperative Oncology Group performance status.^9^ For patients who express programmed death ligand-1 (PD-L1) and are ineligible for cisplatin chemotherapy or any patient not eligible for a platinum-based regimen, antibodies against programmed death-1 receptor (PD-1) or PD-L1 are treatment options.^10^ In patients with mUC, objective response rates (ORRs) for currently approved anti–PD-(L)1 therapies in the second-line setting range from 13% to 21%, with a lower response rate in visceral sites.^10^

EV-101 (ASG-22CE-13-2) is a phase I, dose escalation/dose expansion study in patients with Nectin-4–positive tumors (including mUC) who have previously been treated with ≥ 1 prior chemotherapy regimen. Primary objectives were the determination of safety/tolerability, recommended phase II dose (RP2D), and pharmacokinetic (PK) profile of EV. A secondary objective was to evaluate EV antitumor activity, including confirmed investigator-assessed ORR (RECIST version 1.1), duration of response (DoR), progression-free survival (PFS), and overall survival (OS). In an expansion cohort (part C) of patients with mUC previously treated with anti–PD-(L)1 therapy, response was evaluated by investigator and central radiologic review.

## METHODS

North American patients with Nectin-4–positive solid tumors, including mUC, who progressed on ≥ 1 prior chemotherapy regimen or who were ineligible for cisplatin chemotherapy were enrolled in this open-label, 3-part, dose escalation/dose expansion phase I study. Although Nectin-4 expression was initially a requirement for study enrollment, almost all screened urothelial tumor biopsy samples exhibited the presence of high levels of Nectin-4 by immunohistochemistry (IHC) using an anti-Nectin-4 antibody (clone M22-321b41.1). Because the majority of patients with mUC exhibited high levels of Nectin-4 tumor staining, the protocol was amended, and this eligibility requirement was removed. Additional methodologies for IHC staining and H-scoring of tumor biopsy samples, as well as additional inclusion/exclusion criteria, can be found in the Data Supplement (online only).

In part A, patients with histologically confirmed malignant solid tumors expressing Nectin-4, resistant or refractory to treatment, were enrolled while following a modified continual reassessment method dose escalation design. When safe dose levels were identified, dose levels of interest in part A were expanded for safety and tolerability assessment. After RP2D was established in part A, parts B and C were enrolled. Part B is evaluating EV in 3 dose expansion cohorts, including patients with mUC with severe renal insufficiency, patients with non–small-cell lung cancer, and patients with ovarian cancer. Part C was a dose expansion cohort in patients with mUC previously treated with anti–PD-(L)1 therapy. For this study, anti–PD-(L)1 therapy included, but was not limited to, atezolizumab, pembrolizumab, durvalumab, avelumab, and nivolumab. Because part B was still enrolling at the time of this writing, this article focuses specifically on the results from parts A and C; the full design of the EV-101 study is shown in the Data Supplement.

During part A, patients received increasing weight-based doses (0.5, 0.75, 1.0, and 1.25 mg/kg) through 30-minute infusion on days 1, 8, and 15 of a 28-day cycle. The decision to terminate escalation was determined using dose-limiting toxicity (DLT) boundary limits while following the Pocock stopping rule^11^ (Data Supplement). The DLT rate was determined using a modified continual reassessment method on an ongoing basis; maximum tolerated dose was defined as the dose with a DLT rate of 20%.

Patients who previously progressed on anti–PD-(L)1 therapies prospectively enrolled in part C were treated with EV 1.25 mg/kg on the same schedule used in part A. Patients continued treatment until disease progression or discontinuation as a result of an adverse event (AE), investigator decision to terminate therapy, or consent withdrawal.

Primary objectives were determining the EV safety/tolerability and PK profiles; antitumor activity was a secondary objective. Safety/tolerability end points were based on the rate of DLTs and AEs and were assessed by medical history, physical examinations, clinical laboratory evaluations, and ECG monitoring; AEs were graded using National Cancer Institute Common Terminology Criteria for Adverse Events (version 4.03).

Blood samples for PK assessment were collected after the first and third EV dose administrations. Three analytes were measured: total antibody (T_Ab_), ADC, and MMAE. ADC and T_Ab_ were measured by enzyme-linked immunosorbent assay and validated by Intertek (San Diego, CA). A liquid chromatography-mass spectrometry method was used by Covance (Madison, WI) to measure unconjugated MMAE concentrations in patient plasma samples.

Disease assessment was performed every 8 weeks (± 7 days) with computed tomography/magnetic resonance imaging by the investigator and by a central third-party imaging reviewer (part C only) according to RECIST version 1.1. If a complete response (CR) or partial response (PR) was assessed by the investigator in patients with mUC previously treated with anti–PD-(L)1 therapy, additional scans for confirmation were conducted.

Physical examinations and blood samples for clinical laboratory assessments were collected in cycle 1 and all subsequent cycles on days 1, 8, and 15. Tumor tissue biopsy samples were collected at screening, formalin fixed and paraffin embedded, and assessed centrally by Q^2^ Solutions (Teterboro, NJ) for Nectin-4 protein levels by IHC (Data Supplement).

All AEs and clinical laboratory data were summarized using descriptive statistics. PK data were analyzed by noncompartmental methods for all analytes.

Median OS, median PFS, best ORR (CR + PR), disease control rate (DCR; CR + PR + stable disease [SD]), and median DoR were summarized with 95% CIs by the Clopper-Pearson method for binomial outcomes or by Kaplan-Meier estimation for time-to-event variables. Because the evaluation of antitumor activity was a secondary objective, no statistical hypothesis tests were designed or planned.

This study was designed by the sponsors in collaboration with the investigators and was conducted in accordance with the Declaration of Helsinki and Good Clinical Practice guidelines, principles of informed consent, and requirements of public registration of clinical trials. In addition, the research protocol was approved by each site’s institutional review board, independent ethics committee, or research ethics board, and informed consent was obtained from all study participants at the time of enrollment.

## RESULTS

Between June 23, 2014, and October 25, 2018, 201 patients with Nectin-4–positive tumors (0.5 mg/kg, n = 2; 0.75 mg/kg, n = 19; 1.0 mg/kg, n = 30; 1.25 mg/kg, n = 150) were enrolled. Twenty-five patients were enrolled in the dose escalation phase, and most primary tumors in these patients were from the bladder, renal pelvis, or ureter (n = 21); however, patients with primary tumors of the lung, ovary, colon, and appendix (n = 1 each) were also enrolled. The majority of enrolled patients (n = 155) had mUC and were treated with EV 1.25 mg/kg (0.5 mg/kg, n = 2; 0.75 mg/kg, n = 14; 1.0 mg/kg, n = 27; 1.25 mg/kg, n = 112).

Nectin-4 expression, as determined by IHC H-score, was high in the majority of samples (median H-score, 290; range, 0-300; 4th percentile H-score, 150). Distribution of Nectin-4 expression was skewed, with only 5 samples having a Nectin-4 H-score of < 150 (Data Supplement). As of October 25, 2018, 13 of 155 patients who were treated with EV 1.25 mg/kg were alive; reasons for discontinuation are listed in the Data Supplement.

Most enrolled patients were male (72%) and white (89%), with a median age of 67 years (range, 24-86 years); 29% of patients had ≥ 3 lines of prior systemic therapy in the metastatic setting (Table 1). Prior therapies included platinum-based chemotherapy (96%), anti–PD-(L)1 treatment (72%), and taxane therapy (35%). Primary tumors were located in the bladder (71%) and upper tract (25%) with urothelial histology (75%). The majority of baseline metastases were in the lung (51%) and liver (39%).

**TABLE 1.**
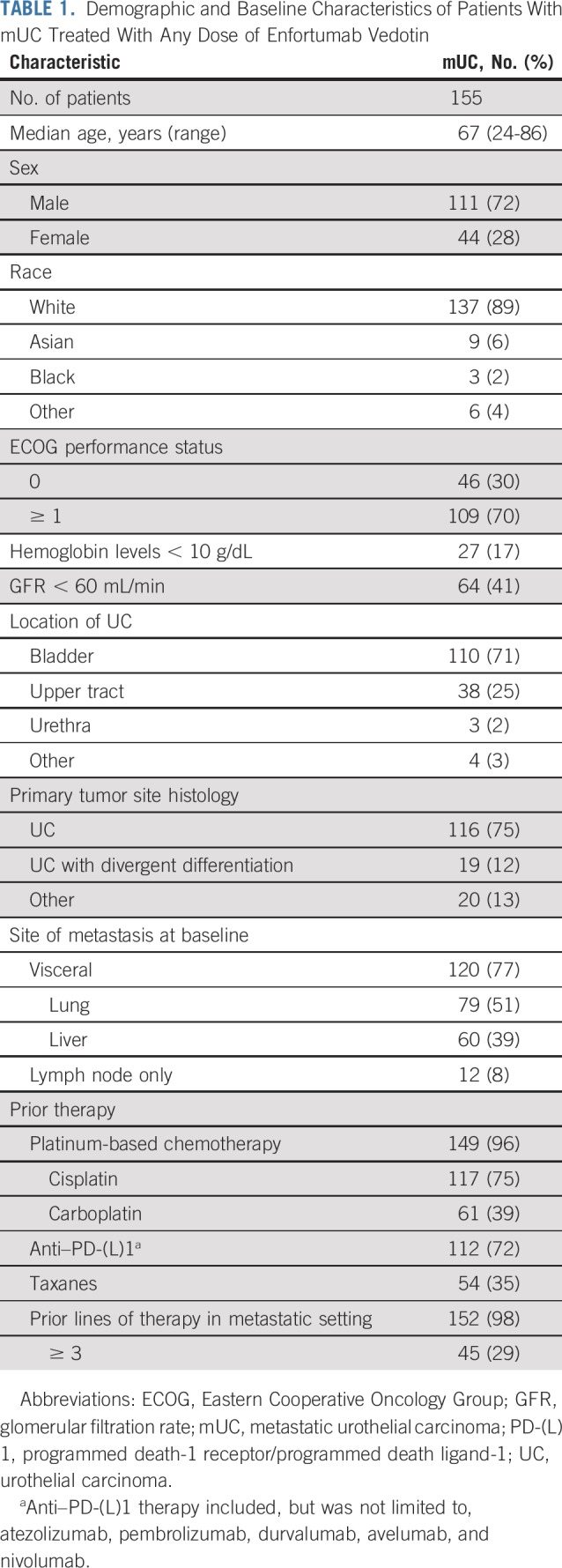
Demographic and Baseline Characteristics of Patients With mUC Treated With Any Dose of Enfortumab Vedotin

Twenty-four patients with Nectin-4–expressing tumors were included in the dose escalation and evaluable for DLTs; of these, 2 (8%) experienced a DLT, both at 1.0 mg/kg. One patient with prior pelvic irradiation experienced grade 2 proctalgia; the other experienced an increase in blood uric acid (grade 4) with no other clinical sequelae. Patients were not assessed for DLTs during dose expansion in part A; however, safety/tolerability continued to be closely monitored.

Of the 155 patients with mUC, 145 (94%) experienced ≥ 1 AE considered at least possibly related to EV (Table 2). The most common treatment-related AEs (TRAEs) that occurred in ≥ 30% of patients receiving 1.25 mg/kg EV were fatigue (53%), alopecia (46%), decreased appetite (42%), dysgeusia (38%), nausea (38%), peripheral sensory neuropathy (38%), pruritus (35%), and diarrhea (33%). Because of the moderate expression of Nectin-4 in normal human skin, rash was an anticipated on-target toxicity. Treatment-emergent rash (of any form) occurred in 70 patients (45%; Data Supplement), and maculopapular rash in 33 patients (21%) was the most commonly reported type of treatment-related rash. Most events of rash were mild or moderate in severity, with 10 patients experiencing a grade 3 rash (maculopapular, n = 5; erythematous, n = 2; eczema, n = 1; palmar-plantar erythrodysesthesia syndrome, n = 1) and 1 patient experiencing a grade 4 bullous dermatitis. For grade 3 rash events, treatment was held until improvement to grade 1. Peripheral neuropathy (of any form), believed to be mediated through the microtubule inhibitor MMAE occurred in 76 patients (49%), with the majority of events grade ≤ 2. Peripheral sensory neuropathy was a commonly reported TRAE that occurred in 49 patients (32%). One patient experienced a grade 3 sensory neuropathy. For events of grade 2-3 neuropathy, treatment was held until improvement to grade 1. Peripheral sensory neuropathy was the most common reason for discontinuation as a result of an AE (5 of 16 patients).

**TABLE 2.**
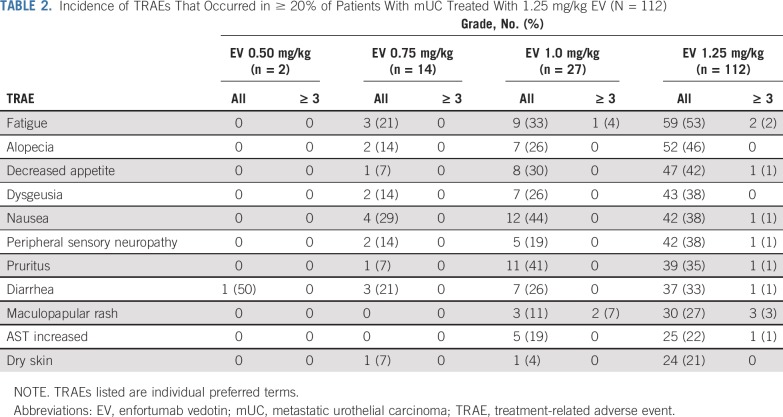
Incidence of TRAEs That Occurred in ≥ 20% of Patients With mUC Treated With 1.25 mg/kg EV (N = 112)

Among the 155 patients with mUC, grade ≥ 3 TRAEs occurred in 53 (34%); hyperglycemia (5%) was the only grade ≥ 3 TRAE that occurred in ≥ 5% of patients. TRAEs that led to discontinuation occurred in 16 patients (10%), with the most common being peripheral sensory neuropathy (n = 5; 3%). Four fatal TRAEs were reported with 1.25 mg/kg in part C (respiratory failure, urinary tract obstruction, diabetic ketoacidosis, and multiorgan failure); however, all events were complex with multiple confounding factors.

It was decided to not evaluate EV at doses > 1.25 mg/kg because of the emergence of drug-related rash and diarrhea (Table 2) and the frequency of dose reductions as a result of AEs, both of which occurred at higher frequencies relative to patients treated with lower doses. While none of the patients with mUC receiving the 0.75 mg/kg dose required a dose reduction because of an AE, 3 (11%) of 27 patients who received the 1.0 mg/kg dose required a dose reduction because of an AE. Thirty-nine (35%) of the 112 patients with mUC treated with 1.25 mg/kg required a dose reduction. RP2D for EV was established as 1.25 mg/kg on days 1, 8, and 15 of a 28-day cycle on the basis of the benefit/risk ratio of antitumor activity, safety, and tolerability results.

The PK profiles of intact ADC, T_Ab_, and MMAE in parts A and C are presented in the Data Supplement. In part A, mean exposure (ie, area under the concentration-time curve [AUC]) of ADC, T_Ab_, and MMAE generally increased with ascending dose. Across the dose range, maximum ADC concentrations were attained approximately 0.5 to 1 hour after intravenous (IV) administration, and intracycle accumulation of ADC was minimal, as suggested by the ratio of partial AUC from days 0-7 after the third and first doses (accumulation ratio [R_AC_], 1.2). Total antibody was generally higher than corresponding intact ADC concentrations with dose-proportional increases of maximal concentration and AUC. The time to reach maximum MMAE concentration was longer than for ADC (approximately 1-2.8 days after IV administration). Intracycle accumulation of MMAE was also minimal, as suggested by the R_AC_ (median, 1.1-1.5). Compared with the EV 1.25 mg/kg PK profile observed in part A, the PK profile observed in part C was not considerably different.

**FIG 1.**
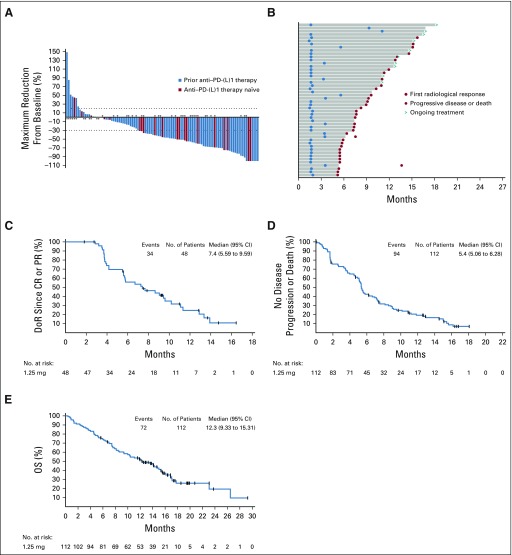
Antitumor effects of enfortumab vedotin 1.25 mg/kg in patients with metastatic urothelial carcinoma. (A) Change in tumor burden from baseline (investigator assessed). A total of 103 patients had at least 1 measurable postbaseline response assessment. (B) Time to response and duration of response (DoR; investigator-assessed responders only). (C) DoR (investigator assessed). (D) Progression-free survival (investigator assessed). (E) Overall survival (OS; investigator assessed). (*) Patients with liver metastasis at baseline. CR, complete response; PD-(L)1, programmed death-1 receptor/programmed death ligand-1; PR, partial response.

Of the 112 patients with mUC treated with EV 1.25 mg/kg, confirmed ORRs were observed in 48 patients (43%; 95% CI, 33.6% to 52.6%; Table 3; Figs 1A and 1B) per investigator assessment; 5 patients (5%) achieved CR and 43 (38%) achieved PR. As of October 25, 2018, the median DoR across these patients was 7.4 months (95% CI, 5.6 to 9.6 months; Fig 1C). Even at doses < 1.25 mg/kg, clinical responses were observed (Data Supplement); ORRs ranged from 18.5% (n = 5 of 27; 1.0 mg/kg) to 50% (n = 1 of 2; 0.5 mg/kg). In addition, 2 (40%) of the 5 patients with low Nectin-4 expression (median H-score, < 150) achieved PR.

**TABLE 3.**
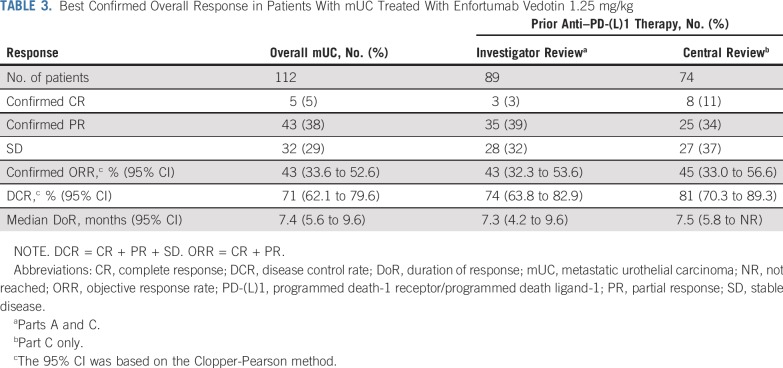
Best Confirmed Overall Response in Patients With mUC Treated With Enfortumab Vedotin 1.25 mg/kg

Across the 112 patients with mUC treated with 1.25 mg/kg EV, 45 had liver metastasis at baseline. Sixteen of these patients (36%) achieved PR. Clinical response rates in anti–PD-(L)1 treatment-naïve patients (n = 10 of 23; 43.5%), patients with prior anti–PD-(L)1 treatment (n = 38 of 89; 42.7%), and patients with prior taxane treatment (n = 11 of 33; 33.3%) were consistent with the overall population (Data Supplement). Similar response rates were observed in patients ≥ 75 years of age (n = 8 of 21; 38.1%) and patients with upper-tract disease (n = 10 of 21; 47.6%).

**FIG 2.**
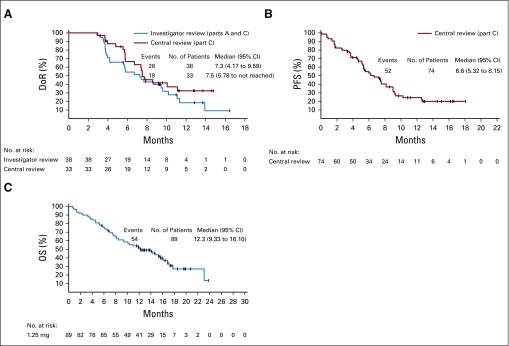
Survival with enfortumab vedotin (EV) 1.25 mg/kg in patients with metastatic urothelial carcinoma (UC) with prior programmed death-1 receptor/programmed death ligand-1 [PD-(L)1] exposure. (A) Duration of response (investigator-assessed responders and central review). (B) Progression-free survival (PFS; central review). (C) Overall survival (OS; investigator assessed).

To ensure unbiased results, patients with prior anti–PD-(L)1 exposure were assessed by both investigator (n = 89, parts A and C) and central review (n = 74, part C). Similar ORRs were observed in these patients (Table 3): 43% (95% CI, 32.3% to 53.6%) by investigator review and 45% (95% CI, 33.0% to 56.6%) by central review. The median DoR for these patients (investigator-assessed review) was 7.3 months (95% CI, 4.2 to 9.6 months); median DoR was 7.5 months (95% CI, 5.8 months to not reached) by central review (Fig 2A).

As of October 25, 2018, 40 (36%) of 112 patients with mUC who were treated with EV 1.25 mg/kg were still alive at a 16.4-month follow-up. The estimated median PFS for these patients was 5.4 months (95% CI, 5.1 to 6.3 months; Fig 1D). As estimated by central review, PFS for patients with prior anti–PD-(L)1 treatment was 6.6 months (95% CI, 5.3 to 8.2 months; Fig 2B).

Median OS with EV 1.25 mg/kg was estimated as 12.3 months (95% CI, 9.3 to 15.3 months); the OS rate at 1 year was 51.8%, with a median follow-up of 16.4 months (Fig 1E). Median OS in the 89 patients with prior anti–PD-(L)1 treatment was estimated as 12.3 months (95% CI, 9.3 to 16.1 months; Fig 2C). Survival estimates for the study subpopulations are presented in the Data Supplement.

## DISCUSSION

This phase I, dose escalation/dose expansion study of EV, a Nectin-4–targeted ADC, identified 1.25 mg/kg administered on days 1, 8, and 15 of a 28-day cycle as the RP2D on the basis of the dose at which EV was active and generally well tolerated. In this study, EV demonstrated single-agent antitumor activity in patients with mUC regardless of prior therapy. These findings with EV are especially encouraging for patients with mUC who had previously received an anti–PD-(L)1 therapy because there are no currently approved treatments. In addition, because of high Nectin-4 expression in urothelial tumor biopsy samples,^12^ prescreening of Nectin-4 expression in tumor samples before EV administration is not necessary, which eliminates diagnostic barriers for EV administration in UC.

In patients with mUC, EV exhibited linear PK from 0.5 to 1.25 mg/kg. Circulating MMAE levels seem to be low relative to ADC, with plasma exposures of MMAE accounting for < 0.1% of ADC serum exposure. Throughout the duration of the study, the most common AEs related to EV were fatigue, alopecia, rash (of any form), peripheral neuropathy (of any form), decreased appetite, dysgeusia, nausea, pruritus, and diarrhea; the majority of AEs considered related to EV were mild to moderate in severity. Only 2 patients (both treated with 1.0 mg/kg EV) experienced a DLT event. Correlation between dose level and the requirement for dose reductions suggested that a higher rate of AEs would be observed at higher dose levels; as such, EV was not escalated beyond 1.25 mg/kg.

Because Nectin-4 has mild to moderate expression on human skin keratinocytes and skin appendages, rash, alopecia, pruritus, and dry skin are likely to be on-target toxicities. Peripheral neuropathy (of any form), believed to be mediated through the microtubule inhibitor MMAE, was observed in 49% of patients. Most events were sensory in nature and mild to moderate in severity; neuropathy led to treatment discontinuation in 3% of patients. Serious cases of hyperglycemia considered related to EV were reported in 3.2% of patients with mUC and 4.8% of patients previously treated with anti–PD-(L)1 therapy. Out of abundance of caution, these cases led to a protocol amendment to exclude patients with uncontrolled diabetes and hold EV dosing for grade ≥ 3 blood glucose levels.

Single-agent EV 1.25 mg/kg provided an encouraging and notable response rate of 43% in patients with mUC; in part A, ORR was 50% (0.5 mg/kg), 21% (0.75 mg/kg), and 18.5% (1.0 mg/kg). A response rate of 45% was observed for patients with mUC previously treated with an anti–PD-(L)1 agent and 36% in patients with baseline liver metastasis. The ORR was also consistent across subgroups of interest, such as patients naïve to anti–PD-(L)1 therapy, patients with upper-tract disease, and patients ≥ 75 years of age. In this study, the estimated median OS was 12.3 months for EV; previously reported OS for anti–PD-(L)1 therapy was up to 10.3 months in patients after platinum-based chemotherapy.^13,14^ Responses in this population seemed durable, with a median DoR of 7.3 months in patients treated with prior anti–PD-(L)1 therapy as assessed by the investigator.

Patients with mUC are in need of additional treatment options, especially those who have progressed after anti–PD-(L)1 therapy where there is no current standard of care. Our study identified single-agent EV as tolerable with antitumor activity in pretreated patients with mUC, including patients with prior anti–PD-(L)1 treatment and patients with liver metastasis at baseline. Because of the strength of these data, single-agent EV has been investigated in patients with locally advanced/mUC previously treated with anti–PD-(L)1 therapy in a pivotal phase II study (EV-201; ClinicalTrials.gov identifier: NCT03219333)^15^ and is currently enrolling a confirmatory randomized phase III study (EV-301; ClinicalTrials.gov identifier: NCT03474107). In addition, EV is being evaluated as a single agent or in combination with an immune checkpoint inhibitor and/or chemotherapy in patients with locally advanced/mUC, including cohorts of patients with muscle-invasive UC (ClinicalTrials.gov identifier: NCT03288545).

## DATA SHARING STATEMENT

Studies conducted with product indications or formulations that remain in development are assessed after study completion to determine whether individual participant data (IPD) can be shared. The plan to share IPD is based on the status of product approval or termination of the compound in addition to the other study-specific criteria described at www.clinicalstudydatarequest.com under sponsor-specific details for Astellas Pharma.
